# Valorisation of Cardoon Leaves and Lemon Peel for Potential Active Food Packaging: Impact of Drying on Nutritional Profile and Functional Performance of Whey Protein Films

**DOI:** 10.3390/foods15183229

**Published:** 2026-09-12

**Authors:** Cássia H. Barbosa, Mariana A. Andrade, Victor G. L. Souza, Francisco Ravasco, Carla Motta, Miguel A. Cerqueira, Vasco D. F. Martins, Andreia F. M. Santos, Sidney Tomé, Fernanda Vilarinho, Ana Sanches Silva, Ana Luísa Fernando

**Affiliations:** 1 Department of Food and Nutrition, National Institute of Health Doutor Ricardo Jorge, Av. Padre Cruz, 1649-016 Lisbon, Portugal; cassia.barbosa@insa.min-saude.pt (C.H.B.); mariana.andrade@insa.min-saude.pt (M.A.A.); francisco.ravasco@insa.min-saude.pt (F.R.); carla.motta@insa.min-saude.pt (C.M.); sidney.tome@insa.min-saude.pt (S.T.); fernanda.vilarinho@insa.min-saude.pt (F.V.); 2 MEtRICs, Department of Chemistry, NOVA School of Science and Technology, NOVA University of Lisbon, Campus de Caparica, 2829-516 Caparica, Portugal; v.souza@fct.unl.pt (V.G.L.S.); afm.santos@fct.unl.pt (A.F.M.S.); 3 REQUIMTE/LAQV, Rua D. Manuel II, Apartado 55142, 4051-401 Porto, Portugal; 4 International Iberian Nanotechnology Laboratory, Av. Mestre José Veiga, 4715-330 Braga, Portugal; miguel.cerqueira@inl.int (M.A.C.); vasco.martins@inl.int (V.D.F.M.); 5 Centre of Biological Engineering, University of Minho, Campus de Gualtar, 4710-057 Braga, Portugal; 6 LAQV-REQUIMTE, Department of Chemistry, NOVA School of Science and Technology, NOVA University of Lisbon, Campus de Caparica, 2819-516 Caparica, Portugal; 7 i3N/CENIMAT, Department of Materials Science, NOVA School of Science and Technology, NOVA University of Lisbon, 2829-516 Caparica, Portugal; 8 Centre for Study in Animal Science (CECA), Instituto de Ciências, Tecnologias e Agroambiente (ICETA), University of Porto, 4050-053 Porto, Portugal; asanchessilva@ff.uc.pt; 9 Faculty of Pharmacy, University of Coimbra, Azinhaga de Santa Comba, 3000-548 Coimbra, Portugal; 10 Associate Laboratory for Animal and Veterinary Sciences (AL4AnimalS), 1300-477 Lisbon, Portugal

**Keywords:** *Cynara cardunculus* L., by-products, whey protein concentrate, dietary fibre

## Abstract

Despite being usually discarded, agro-industrial by-products are rich in bioactive and nutritional compounds and can be redirected into new foods or food packaging. This study evaluated the nutritional composition of cardoon leaves (*Cynara cardunculus* L.), an agro-industrial by-product, and assessed the impact of the drying process on those characteristics. Furthermore, extracts of the dried leaves and dried leaves combined with lemon peel were incorporated into whey protein-based films at concentrations of 0.5%, 1.0%, and 2.0% (*w*/*v*). Their physical, barrier, mechanical, and thermal properties were also analysed. When compared on a dry weight (DW) basis, the dry leaves maintained a stable nutritional profile with no significant degradation of key components, presenting with high carbohydrates (15.4 ± 1.1 g/100 g), moderate protein (21.2 ± 0.4 g/100 g), and low fat (2.0 ± 0.1 g/100 g). The leaves were also a good source of dietary fibres (43.9 ± 1.8 g/100 g) and minerals (13.7 ± 0.2 g/100 g of ash). Incorporating the extracts altered the films’ physicochemical properties, reducing their water vapour permeability up to 4.05 ± 0.26 10^−10^ g/s.m.Pa, while their tensile strength decreased from 0.34 ± 0.06 to 0.23 ± 0.01 MPa depending on the formulation, and their elongation at break decreased by up to 6.58 ± 0.70%. In contrast, Young’s modulus increased by up to 0.11 ± 0.03 MPa, indicating higher rigidity. This behaviour may be attributed to the interactions between the phenolic compounds and the whey protein matrix, which restricted polymer chain mobility and increased film rigidity while reducing matrix cohesion and flexibility. The results demonstrate that while the extracts enhanced the barrier properties of the films, they reduced the mechanical properties. Overall, the results suggest that cardoon by-products have potential applications in active food packaging, contributing to the development of bio-based materials within a circular economy framework.

## 1. Introduction

In the agro-industrial sector, large amounts of by-products are generated daily, even though they are rich in nutrients (e.g., fibre, protein, and minerals) and phytochemical compounds (e.g., phenolics, terpenoids and carotenoids). Despite being normally discarded, these by-products can improve nutritional properties of and provide functional benefits to foods, and be used in the development of new active food packaging [1,2].

*Cynara cardunculus* L. (cardoon) is a Mediterranean multipurpose crop, with the leaves being the main by-product generated from the usage of the flowers as vegetable rennet in cheese making [3,4]. Several studies have described the polyphenol profile of cardoon leaves [5,6,7,8,9], but despite its diverse applications, few studies have reported their nutritional composition [9]. Due to their composition, cardoon leaves can be used as an active compound in the development of new biodegradable food packaging; however, the impact of processes like drying on their composition is a critical factor for their valorisation.

In parallel, whey protein, also a by-product of the cheese industry, can be used as a biopolymer in the production of biodegradable films and/or coatings in the food industry. Whey protein produces a film/coating that is normally transparent, colourless, odourless, and flexible, with adequate water vapour permeability and, when combined with an active compound, can help to prolong foods’ shelf life [10,11]. Numerous researchers have produced whey protein films and coatings with different active compounds [12,13,14,15,16,17,18,19,20,21]. For example, Andrade et al. [12] developed a whey protein film incorporating F. vesiculosus extract, which increased the tensile strength and elastic modulus of the film, decreased its elongation, and increased the water vapour permeability. In addition, the active film was able to prolong chicken’s shelf life, as it delayed its lipid oxidation. Additionally, in the study of Ming et al. [21], the incorporation of tea polyphenols into a whey protein coating enhanced its antioxidant properties, which was further confirmed by the reduction in the browning and weight loss of apple slices during storage.

Lemon peel is another abundant agro-industrial waste well known for its high bioactive profile, such as flavonoids and essential oils, which provide significant antioxidant and antimicrobial properties [22]. The application of lemon peel, alone or in combination with other extracts, can reinforce a polymer matrix by improving its functional properties [22,23].

Even with the potential of cardoon leaves and the known benefits of citrus by-products, further characterisation of cardoon leaves is needed to support their valorisation as a potential food and packaging resource. Additionally, despite the multiple studies that have used different plant extracts in whey protein films, the combination of cardoon leaf extract and lemon peel extract has not yet been used in the development of active food films/coatings.

Hence, this study aims to address these gaps by providing a detailed characterisation of the nutritional composition of cardoon leaves, including their amino acid, fatty acid, and carotenoid profiles, while evaluating the impact of drying on these components, a simple, low-cost, and widely applicable preservation method. Moreover, following sustainability and circular economy principles, this study aims to develop and characterise whey protein-based films incorporating cardoon leaf extract at varying concentrations (0.5%, 1.0%, and 2.0%). In addition, lemon peel extract is also incorporated based on its reported bioactive properties and to evaluate its effect, in addition to that of cardoon leaves, on the resulting film properties. The resulting films are evaluated for their physical, barrier, mechanical, structural, and thermal properties, transforming underutilised agro-industrial waste into an innovative and robust material for food packaging.

## 2. Materials and Methods

### 2.1. Sample Preparation

The *Cynara cardunculus* var. *altilis* DC. (cultivated cardoon) leaves were kindly supplied by a local producer from Miguel Choco in the Guarda Region, Portugal. The leaves were collected by the producers during the spring–summer of 2024. At the laboratory, the leaves were washed and separated from the stems. Afterwards, the leaves were separated in two groups:

• Group one—fresh leaves: The leaves did not suffer any kind of treatment; they were just ground, vacuum packaged, stored at 4 ± 2 °C, and protected from light until use.
• Group two—dry leaves: The leaves were dried in an oven with forced air circulation at 35 °C for 48 h until a constant weight was reached, corresponding to a moisture content of less than 10%; they were then ground, vacuum packaged, stored at room temperature (±21 °C), and protected from light until use.

The lemon peels were kindly supplied by a Portuguese juice company, Frubaça-Cooperativa de Hortofruticultores, Leiria, Portugal. Upon their arrival at the laboratory, the by-products were frozen and freeze-dried, ground, vacuum packaged, stored at room temperature (±21 °C), and protected from light until use.

### 2.2. Nutritional Profile of Fresh and Dry Cardoon Leaves

#### 2.2.1. Proximate Composition Profile

The fresh and dry cardoon leaves were analysed for their moisture, ash, total protein, total fat, total dietary fibre and total sugars contents. The carbohydrate contents and energy values were determined by calculation. The moisture content was analysed gravimetrically using a dry air oven at 100 ± 2 °C (Memmert, Schwabach, Germany) [24]. The ash content was assessed by incineration of the samples in a muffle furnace (525 ± 25 °C; 20 h) [24]. The protein content was determined by the Kjeldahl method, quantifying the total nitrogen present in the samples by using a block digestion system, a Foss Tecator 2006 Digestor and a Foss 2200 Kjeltec Auto Distillation unit (Foss, Hilleroed, Denmark) [24]. The total fat content was determined using the Soxhlet extraction method (Soxtec™ 2050, Foss, Hilleroed, Denmark) with acid hydrolysis, and using petroleum ether as the extraction solvent [24]. The total dietary fibre was determined using an enzymatic–gravimetric method [24]. The determination of the total sugar was assessed by the Munson and Walker method [25]. All assays were done in triplicate and the results are presented as mean ± standard deviation.

#### 2.2.2. Fatty Acid Profile

The extraction and methylation of fatty acids followed the WHO’s simplified protocol [26] for quantifying trans-fatty acids as a percentage of the total fatty acids in food products. Briefly, a sample was mixed with petroleum ether, followed by phase separation and filtration through anhydrous sodium sulphate. The solvent was then evaporated by rotary evaporation (Büchi model R-210, Labortechnik, Wettingen, Switzerland) under controlled conditions (40 °C). Following solvent evaporation, the extracted fat was methylated with *n*-heptane and methanolic KOH and the clear upper phase was then collected for the GC-FID analysis using an Agilent 7890A Gas Chromatograph (Santa Clara, CA, USA) equipped with an FID detector, and an SP2380 column (100 m × 250 µm × 0.2 µm) from Supelco (Milan, Italy) was used to identify and quantify the fatty acids. Helium was the carrier gas, at a constant flow rate of 1.2 mL/min, linear velocity of 20 cm/s, and a column head pressure of 230 kPa. The injection volume was 1 μL in split mode (100:1), with the injector and detector temperatures set at 260 °C and 270 °C, respectively. The oven programme was initial temperature of 60 °C, hold 1 min; ramp at 8 °C/min to 100 °C, hold 10 min; ramp at 4 °C/min to 180 °C, hold 20 min; and ramp at 2 °C/min to 215 °C, hold 32 min (106 min total). The certified reference material consisted of 37 FAMEs, ranging from C4 to C24 (Sigma-Aldrich, St. Louis, MO, USA), prepared in *n*-heptane at 10 mg/mL and stored in aliquots at −20 °C. The assay was done in triplicate and the results are presented as mean ± standard deviation.

#### 2.2.3. Amino Acid Profile

For the amino acid profile determination [27], about 25 mg of sample was weighed into a quartz digestion vial and 200 µL of an internal standard solution (25 mM D-Norvaline) and 1 mL of hydrochloric acid (6 N) with 0.5% phenol were added. A closed-system microwave digestion device (Milestone ETHOS 1 Series, Milestone, Sorisole, Italy) was used to digest the samples (15 min to increase the temperature to 160 °C, 10 min at 160 °C and 90 min to cool). Then, 1 mL of sodium hydroxide (6 N) was added to neutralise the samples following hydrolysis. Ultrapure water was added to the system to achieve a final volume of 10 mL and then filtered. To perform the derivatisation procedure (55 °C, 10 min), 80 µL of borate buffer, 20 µL of derivatisation reagent (6-aminoquinolyl-N-hydroxysuccinimidyl carbamate), and 10 µL of sample were mixed in a chromatography vial.

A Waters ACQUITY™ UPLC system (Waters Corporation Company, Milford, MA, USA) with a photodiode array detector (PDA) and an ACQUITY™ UPLC BEH C18 column (100 mm × 2.1 mm, 1.7 µm) was used for the analysis. A flow rate of 0.7 mL/min was defined, and the column temperature was set at 55 °C. Two eluents—AccQTag ultra eluent A diluted in 95% ultrapure water and AccQTag ultra eluent B—from Waters Corporation Company constituted the mobile phase. The elution gradient was set as follows: 0–0.54 min, 99.9% A–0.1% B; 5.74 min, 90.9% A–9.1% B; 7.74 min, 78.8% A–21.2% B; 8.04 min, 40.4% A–59.6% B; and 8.70–10 min, 99.9% A–0.1% B. The injection volume was 1 µL, and the samples were monitored at 260 nm. Empower™ software version 2.0 (Waters, Milford, MA, USA) was used to process and quantify the peak areas.

#### 2.2.4. Mineral Profile

The mineral profile was determined according to Nascimento et al. [28]. Initially, an acid digestion of the samples was performed, during which approximately 0.5 g of each sample was accurately weighed into Teflon digestion vessels. A solution consisting of concentrated nitric acid (4 mL), hydrogen peroxide (1 mL), and deionised water (3 mL) was then carefully added. The vessels were tightly sealed and subjected to microwave-assisted digestion using a closed-vessel system (Milestone ETHOS 1 Series). Digestion was carried out following a previously optimised five-step program, with temperatures ranging from 90 °C to 210 °C and durations between 6 and 12 min. After completion, the vessels were allowed to cool to room temperature before being opened. The digests were then diluted to a final volume of 25 mL with deionised water and transferred into analytical tubes. Elemental analysis of copper (Cu), manganese (Mn), iron (Fe), zinc (Zn), magnesium (Mg), calcium (Ca), phosphorus (P), sodium (Na), and potassium (K) was conducted using an inductively coupled plasma optical emission spectrometer (ICP-OES, Thermo iCAP 6000 series, Thermo Scientific, Waltham, MA, USA) operated in both radial and axial modes.

#### 2.2.5. Carotenoids Profile

Carotenoid quantification (α-carotene, β-carotene, β-cryptoxanthin, lycopene, lutein, and zeaxanthin) was carried out using reversed-phase High-Performance Liquid Chromatography (HPLC) with UV/Vis diode array detection [29]. Samples were homogenised and extracted under low-light conditions using a tetrahydrofuran:methanol (1:1, *v*/*v*) solution containing butylated hydroxytoluene (BHT, 0.1%) as an antioxidant. Depending on the lipid content, the extracts were saponified with a 10% KOH methanolic solution to obtain the free carotenoid forms. The organic extracts were evaporated under nitrogen at ≤40 °C and reconstituted in the mobile phase before injection. Chromatographic separation was performed using two analytical columns connected in series: a Waters Spherisorb ODS2 column with PEEK coating, coupled to a reversed-phase column containing polymerically modified C18 silica, or any equivalent column capable of achieving the desired separation performance. Acetonitrile and methanol/dichloromethane mixtures containing ammonium acetate, triethylamine, and BHT (67:33, *v*/*v*) were used as the mobile phase components. Quantification was achieved through external calibration with pure all-trans carotenoid standards. An internal standard recovery (β-apo-8′-carotenal) was used to correct for extraction losses. The results are expressed as mg of carotenoid per gram of sample.

#### 2.2.6. Determination of Vitamin A and Vitamin E

Vitamin A and E levels were determined simultaneously by normal-phase HPLC with fluorescence detection [30,31]. Representative samples were homogenised and immediately analysed due to the light sensitivity of the analytes. Saponification was carried out under nitrogen with methanolic ascorbic acid (0.5%) and 60% KOH solution in a water bath with condensation to prevent oxidation. The unsaponifiable fraction was extracted four times with a diethyl ether/petroleum ether (2:8, *v*/*v*) mixture, washed with water to neutrality, and evaporated under reduced pressure at ≤40 °C. The dry residue was reconstituted in *n*-heptane and filtered through 0.45 µm PVDF filters before HPLC analysis. Separation was achieved on a normal-phase silica column (Lichrosorb Si60) using *n*-heptane/2-propanol (98:2, *v*/*v*) as the mobile phase. Detection was performed by fluorescence, using excitation and emission wavelengths specific to each vitamin. Quantification was based on calibration curves prepared from pure standards of all-trans retinol, 13-cis retinol, and dl-α-tocopherol. The results are expressed in mg of vitamin per gram of sample.

### 2.3. Preparation of WPC-Based Films

The films were prepared using whey protein concentrate (WPC; Amazin’ Foods, protein content = 78.8 g/100 g) and incorporated dried cardoon leaf extract and an extract combining cardoon dry leaves with lemon peel. The methods used to prepare the extracts and films are described in the following sections. A total of seven different films were prepared: one control whey protein film (without extract—WPC-C); three active films with 0.5%, 1.0% and 2.0% (*w*/*v*) cardoon leaf extract (WPC-0.5C, WPC-1.0C, and WPC-2.0C, respectively); and three active films with 0.5%, 1.0% and 2.0% (*w*/*v*) cardoon leaf and lemon peel extract (WPC-0.5CL, WPC-1.0CL, and WPC-2.0CL, respectively).

#### 2.3.1. Extract Preparation

The extracts were produced using a solid–liquid extraction using a rotary evaporator, and ethanol was used as the solvent [32]. Briefly, in a centrifuge tube, the sample and the solvent were mixed (10% cardoon leaves and 2.5% lemon peel when added), homogenised in a compact stirrer (Edmund Bühler GmbH model KS-15, Hechinger, Villingen-Schwenningen, Germany) for 30 min, and then centrifuged for 10 min at 10 °C and 11,952× *g*. The supernatant was transferred to a pear-shaped amber evaporation flask, and the solvent was evaporated at 35 °C, until dryness, in the rotary evaporator. The extract was collected with the aid of a spatula, vacuum packaged and stored at −20 °C until further use.

#### 2.3.2. Film Preparation

The preparation of the films was done according to Andrade et al. [32]. Briefly, ultrapure water was added to the whey protein concentrate (8% protein, *w*/*w*) and homogenised at 9500 rpm in an Ultra-Turrax for 2 min. The pH was adjusted to 7.0 with 1 M sodium hydroxide (NaOH) solution and the solution was subjected to a thermostatic bath at 80 °C for 30 min. Then, the solution was rapidly cooled to room temperature, and glycerol at 8% (*w*/*w*) was incorporated and homogenised. The films were casted onto a proper glass surface and dried for 48 h at room temperature (23 ± 2 °C). For the active film, the extract was homogenised with glycerol and then added to the solution for further drying for 48 h at room temperature. The dried crude extract was incorporated at concentrations of 0.5%, 1.0%, and 2.0% (*w*/*v*), corresponding to 0.5, 1.0, and 2.0 g of dry extract per 100 mL of film-forming solution, respectively.

### 2.4. Film Characterisation

#### 2.4.1. Physicochemical Properties

##### Thickness

The thickness of the films was measured using a digital micrometre (Mitutoyo, Kawasaki, Japan). The measurements were done at five different points on each sample (three replicates per film), and the results are presented as the average.

##### Moisture Content, Solubility, and Swelling

The moisture content, solubility in water and the swelling degree of the films were determined according to the method described by Souza et al. [33]. The film specimens were cut into squares (2 × 2 cm) and weighed on an analytical balance, obtaining the initial mass (M1); then, the samples were dried at 70 °C for 24 h in a conventional oven and weighed to obtain the dry mass (M2). Subsequently, the samples were placed in Petri dishes containing 30 mL of Milli-Q water and stored for 24 h at room temperature (25 ± 2 °C) to allow the swelling process to occur. After this contact period, the specimens were superficially dried with filter paper and weighed (M3) again. The residual film specimens were dried in an oven at 70 °C for 24 h to determine the final dry mass (M4). The parameters were calculated according to Equations (1)–(3).

(1) % humidity (g100 g of film)=(M1−M2)M1×100

(2) % solubility (g100 g of film)=(M2−M4)M2×100

(3)               % swelling degree (g100 g of film)=(M3−M2)M2×100

##### Opacity and Transparency

The film opacity and transparency were determined according to Souza et al. [33]. The opacity was obtained by direct reading of the absorbance of rectangular samples at 600 nm using a UV/VIS spectrophotometer (Thermo Scientific Evolution 300 LC, Waltham, MA, USA) and calculated according to Equation (4):

(4) $$Opacity\;({mm}^{-1})=\frac{absorbance\;600\;nm}{sample\;thickness\;(mm)}$$

The film transparency was determined by a spectrum scan (wavelengths between 200 and 900 nm) of each film specimen using the same UV/VIS spectrophotometer. The measurements were performed using air as the reference and the results are expressed as a percentage of transmittance.

##### Colour

The measurement of the CIE-L* a* b* coordinates were used to assess the colour of the film specimens. A CR 410 colorimeter (Minolta Co., Tokyo, Japan) with a D65 light source and a 10-degree visual angle was used for the measurement. The measurements were done on a standardised white background, and five shots were taken at different locations on each film. The results are presented as the average of the individual coordinates (L*, a*, and b* values), chroma C* (Equation (5) and the hue angle h° (Equation (6)).

(5) $$C^{*}={\left(a^{2}+b^{2}\right)}^{0.5}$$

(6) h°=atanb∗a∗

#### 2.4.2. Mechanical Properties

The mechanical properties of the films were measured using a TA.XTplusC Texture Analyser (Stable Micro Systems, Godalming, United Kingdom) equipped with a 500 N load cell according to ASTM D882 with some modifications. Strips of each film (20 mm wide and 80 mm long) were mounted in the tensile grips with an initial gauge length of 50 mm and stretched at a crosshead speed of 12.5 mm/min until breakage. The tensile strength, modulus of elasticity and elongation-at-break were determined from the stress–strain curve.

#### 2.4.3. Barrier Properties

##### Water Vapour Permeability

The measurement of water vapour permeability (WVP) was performed gravimetrically based on ASTM E96/E96M-10 [34]. The permeability cups (Elcometer 5100 Payne Cup, Manchester, UK) were filled with anhydrous calcium chloride. Then, the films were sealed on the top of the cups and placed in a desiccator with controlled relative humidity (50 ± 2%) and temperature (23 ± 2 °C). The cups were periodically weighed at intervals of 1 h for 24 h at ambient temperature. The WVP was calculated according to Equation (7), where *S* is the slope of the straight line of weight gain (g) versus time (s), *A* is the cell area (m^2^), *t* is the film thickness and ∆*P* is the partial pressure difference across the film.

(7) WVP=SA×t∆P

#### 2.4.4. Thermogravimetric Analysis (TGA)

For the thermal characterisation (thermogravimetric analysis (TGA) and differential scanning calorimetry (DSC)), the control film and the films containing 2.0% cardoon and 2.0% cardoon–lemon extracts were selected as representative formulations, as 2.0% was the highest extract concentration evaluated in this study.

The thermal stability of the films was evaluated using a Thermogravimetric Analyser (Setaram Labsys EVO, Caluire-et-Cuire, France) with weighing precision of ±0.01%. The film samples (14–31 mg) were analysed from ~30 °C to 700 °C at a constant heating rate of 10 °C/min, under a continuous argon atmosphere.

#### 2.4.5. Differential Scanning Calorimetry (DSC)

The differential scanning calorimetry (DSC) tests were carried out using a DSC Q2000 from TA Instruments Inc. (Tzero DSC Technology, New Castle, DE, USA) coupled to an RCS 90 cooling system and operating in the Heat Flow T4P mode. Approximately 13 mg of each sample was weighed and encapsulated in a hermetic Tzero aluminium pan and pinholed lid to allow water evaporation. The experimental procedure consisted of first equilibrating all samples at 20 °C, followed by cooling to −90 °C and heating up to 110 °C, keeping the samples at this temperature for 5 min for water evaporation. After this initial cycle, the samples were submitted to an additional cooling and heating cycle between −90 °C and 110 °C. All heating and cooling cycles were performed at a rate of 10 °C/min. The data treatment was carried out with Universal Analysis 2000 software (Version 4.5A) by TA Instruments Inc.

#### 2.4.6. Scanning Electron Microscopy (SEM)

The morphology of films’ surfaces and cross-sections was observed using an SEM (Quanta 650 FEG, FEI, Hillsboro, OR, USA) with an accelerating voltage of +5 kV at different magnifications. The samples were cut with a blade and mounted on sample holders with double-sided adhesive and sputtered with a 10 nm layer of gold using a Leica EM ACE200 (Leica Microsystems GmbH, Wetzlar, Germany).

#### 2.4.7. Attenuated Total Reflectance Fourier-Transform Infrared Spectroscopy (ATR-FTIR)

The attenuated total reflectance Fourier-transform infrared spectroscopy (ATR-FTIR) spectra of the films were obtained using an FTIR spectrometer (PerkinElmer Spectrum Two, Perkin Elmer, Shelton, CT, USA) equipped with a universal attenuated total reflectance sampling accessory. The spectra were collected 4000 to 400 cm^−1^ with 1 cm^−1^ resolution and 32 scans.

### 2.5. Statistical Analysis

The statistical analysis of the results was performed using IBM^®^ SPSS^®^ Statistics software, version 27.0.1.0 (Chicago, IL, USA). A one-way analysis of variance (ANOVA) was conducted, followed by Tukey’s post hoc test to identify significant differences between the mean values. Differences were considered statistically significant at *p* < 0.05. All data are expressed as mean ± standard deviation.

## 3. Results and Discussion

### 3.1. Nutritional Profile of Fresh and Dried Cardoon Leaves

The nutritional composition of fresh and dried cardoon leaves is presented in Table 1. The drying process significantly (*p* < 0.05) reduced the moisture content from 81.3 ± 0.3 g/100 g to 7.4 ± 0.1 g/100 g, a reduction of approximately 90%, confirming the effectiveness of the time–temperature combination applied in the drying oven for the drying of plant material. The drying process can alter the concentration of some nutrient components, by increasing or decreasing them [5,35], as observed in this study (Table 1). The ash content significantly (*p* < 0.05) increased from 12.7 ± 0.0 g/100 g to 13.7 ± 0.1 g/100 g (Table 1), suggesting that the mineral components of the leaves were preserved during the drying process. In both samples, potassium (3668.1 ± 106.6 and 3733.9 ± 63.7 mg/100 g), calcium (1740.7 ± 23.5 and 1665.5 ± 15.1 mg/100 g), and phosphorus (367.8 ± 21.2 and 535.1 ± 9.0 mg/100 g) were present in higher amounts. On the other hand, copper (0.2 ± 0.0 and 0.7 ± 0.0 mg/100 g) and zinc (2.4 ± 0.2 and 3.4 ± 0.1 mg/100 g) were present in low amounts in both the fresh and dry cardoon leaves (Table 1). Potassium (K) was the most abundant mineral in the cardoon leaves and demonstrated thermal stability, with no significant difference between the fresh leaves and dry leaves (3668.1 ± 106.6 and 3733.9 ± 63.7 mg/100 g). In addition, the other minerals analysed by ICP-OES showed statistically significant differences (*p* < 0.05), with increases in the concentrations of P, Mg, Na, Zn, and Cu, and decreases in Ca, Mn, and Fe. The presence of these minerals suggests that cardoon leaves can be incorporated into food products, increasing their nutritional value and functional properties, as these minerals participate in several biochemical processes, like controlling blood pressure and decreasing the risk of cardiovascular problems [36]. The total fat significantly (*p* < 0.05) increased after the drying process, but both the fresh and dried leaves contained low levels of total fat (1.1 ± 0.0 g/100 g and 2.0 ± 0.1 g/100 g), including saturated (SFAs), monounsaturated (MUFAs), and polyunsaturated fatty acids (PUFAs) (Table 1). The increase in fat may be due to the increased rupture and permeability of plant cell membranes. A total of seven fatty acids were identified in the fresh cardoon leaves and 16 in the dry cardoon leaves. The main fatty acids identified were: lignoceric acid (C24:0); cis-8,11,14 eicoastrienoic acid (C20:3 (*n*6)); and γ-linolenic acid (C18:3 (*n*6)). Although there was an increase in the total fat content, levels of certain fatty acids, such as linoleic acid (C18:2c (*n*6)), decreased significantly, likely due to lipid oxidation of the most vulnerable individual unsaturated fatty acids. Both the protein content (22.3 ± 0.6 g/100 g and 21.2 ± 0.4 g/100 g) and total sugars (5.0 ± 0.0 g/100 g and 3.7 ± 0.0 g/100 g) decreased (Table 1) during the drying process, which is a classic biochemical indication of a Maillard reaction [37]. These also may correlate with the decrease in the total energy content (1127 ± 4 KJ/100 g and 1111 ± 9 KJ/100 g; 270 ± 1 Kcal/100 g and 267 ± 2 Kcal/100 g) through the chemical consumption of reducing sugars and the reduction in the bioavailability and digestibility of the protein and carbohydrate fractions. This process demonstrates how different processing methods affect nutrient stability, which can help define strategies for leaf conservation [37]. Regarding the amino acids profile, the individual amino acids significantly (*p* < 0.05) increased or decreased, except for isoleucine and valine (Table 1), indicating thermal degradation and an increase in extractability due to partial hydrolysis. Methionine was the essential amino acid that exhibited the most significant decrease in concentration (433.5 ± 16.7 mg/100 g and 215.8 ± 8.1 mg/100 g) during the drying process. Among the non-essential amino acids, alanine showed the greatest reduction (2343.1 ± 100.4 mg/100 g and 1308.4 ± 4.1 g/100 g). This decrease may be attributed to a Maillard reaction, in which amino acids interact with carbonyl groups from reducing sugars, leading to the consumption of free amino acids. Additionally, general degradation pathways, oxidation, and interactions with other constituents may also contribute to this decrease [37]. On the other hand, lysine was the essential amino acid that exhibited the most significant increase in concentration (458.9 ± 20.6 mg/100 g and 1053.2 ± 40.6 g/100 g) during the drying process, and aspartic acid among the non-essential amino acids (1030.3 ± 47.3 mg/100 g and 3593.6 ± 104.5 mg/100 g). This increase resulted from protein hydrolysis that released amino acids from the structural macromolecular protein complexes, increasing their solubility and chromatographic detection in the analysis of amino acids released after acid hydrolysis [38,39]. Fibre was the major macronutrient found in both samples (42.1 ± 2.5 g/100 g in fresh leaves and 43.9 ± 1.8 g/100 g in dry leaves) (Table 1), and was not significantly (*p* < 0.05) affected by the drying process. This shows that fibre has excellent thermal stability and does not suffer chemical breakdown at conventional drying temperature [40,41]. In plants, the main function of fibre is to provide structure [42], while in human health it plays crucial roles in several chronic diseases, such as cardiovascular diseases, type II diabetes, obesity, colon cancer, and inflammation [43]. Drying the cardoon leaves significantly (*p* < 0.05) decreased the carotenoids analysed, except for *cis*-β-carotene, which showed no significant (*p* < 0.05) variation. The maintenance of the levels of *cis*-β-carotene is possibly due to the thermal isomerisation of *trans*-β-carotene to the *cis* form during the drying process. In contrast, the marked reduction in the other carotenoids analysed indicates greater sensitivity to heat and oxidation [44]. Thus, although drying can be considered an effective method for preserving cardoon leaves, the stability of carotenoids should be carefully evaluated to minimise losses. Regarding vitamins, vitamin A was not detected in any of the samples. Vitamin E showed a decrease in its concentration from 4.92 ± 0.01 mg/100 g to 1.73 ± 0.00 mg/100 g, demonstrating, once again, the sensibility to heat during the drying process for preserving the properties of the leaves.

Although cardoon is used in traditional Mediterranean cuisine [3] and there are several studies characterising its different parts [6,45,46,47], the nutritional data for cardoon leaves remain limited. Chihoub et al. [9] obtained similar results for the fat (1.5 ± 0.1 g/100 g), protein (15.7 ± 0.1 g/100 g), and ash (11.8 ± 0.5 g/100 g), but higher amounts of carbohydrates (71.0 ± 0.5 g/100 g) and energy (360.03 ± 0.5 kcal/100 g) for *Cynara cardunculus* L. var. *sylvestris* (wild cardoon) leaves. The authors also evaluated the fatty acid profile and found α-linolenic acid (C18:3 (*n*3)), linoleic acid (C18:2 (*n*6)), and palmitic acid (C16:0) in higher concentrations [9].

The findings of this study indicate that cardoon leaves present an interesting nutritional profile, especially as a source of dietary fibre, minerals and amino acids. As demonstrated in our previous study [5], cardoon leaves have also shown potential as antioxidant and antimicrobial agents, supporting their applicability as a food ingredient or active compound in bio-based food packaging. In this study, the antioxidant and antimicrobial properties of fresh and dry cardoon leaves were assessed. In general, the dried leaves exhibited higher antioxidant capacity and higher levels of total phenolic compounds and total flavonoids compared to the fresh leaves. In addition, they exhibited greater antimicrobial activity, showing activity against *Staphylococcus epidermidis*, *Enterococcus faecalis*, *Listeria monocytogenes*, *Bacillus cereus*, and *Aspergillus fumigatus* [5]. Similarly, in this present study, a significant difference (*p* < 0.05) was observed between the dry and fresh leaf samples, with the dried leaves exhibiting a better nutritional profile. The drying process proved to be a suitable method to preserve the leaves and their nutritional content, while also supporting the development of novel products that incorporate this by-product as an ingredient, leading to a reduction in agricultural waste while generating value-added products. Therefore, based on its nutritional profile and its previously determined antioxidant and antimicrobial properties [5], only the dried leaves will be used in the preparation of the films.

### 3.2. Film Development and Characterisation

To valorise cardoon leaves, which are typically discarded, whey protein concentrated-based films incorporating dry cardoon leaf extract were produced at three different concentrations (0.5%, 1.0% and 2.0%). Additionally, three whey protein concentrated-based films incorporating dry cardoon leaf–lemon peel extract were also produced at the same concentrations. All films were successfully prepared using the casting method.

#### 3.2.1. Physicochemical Properties

**Figure 1:**
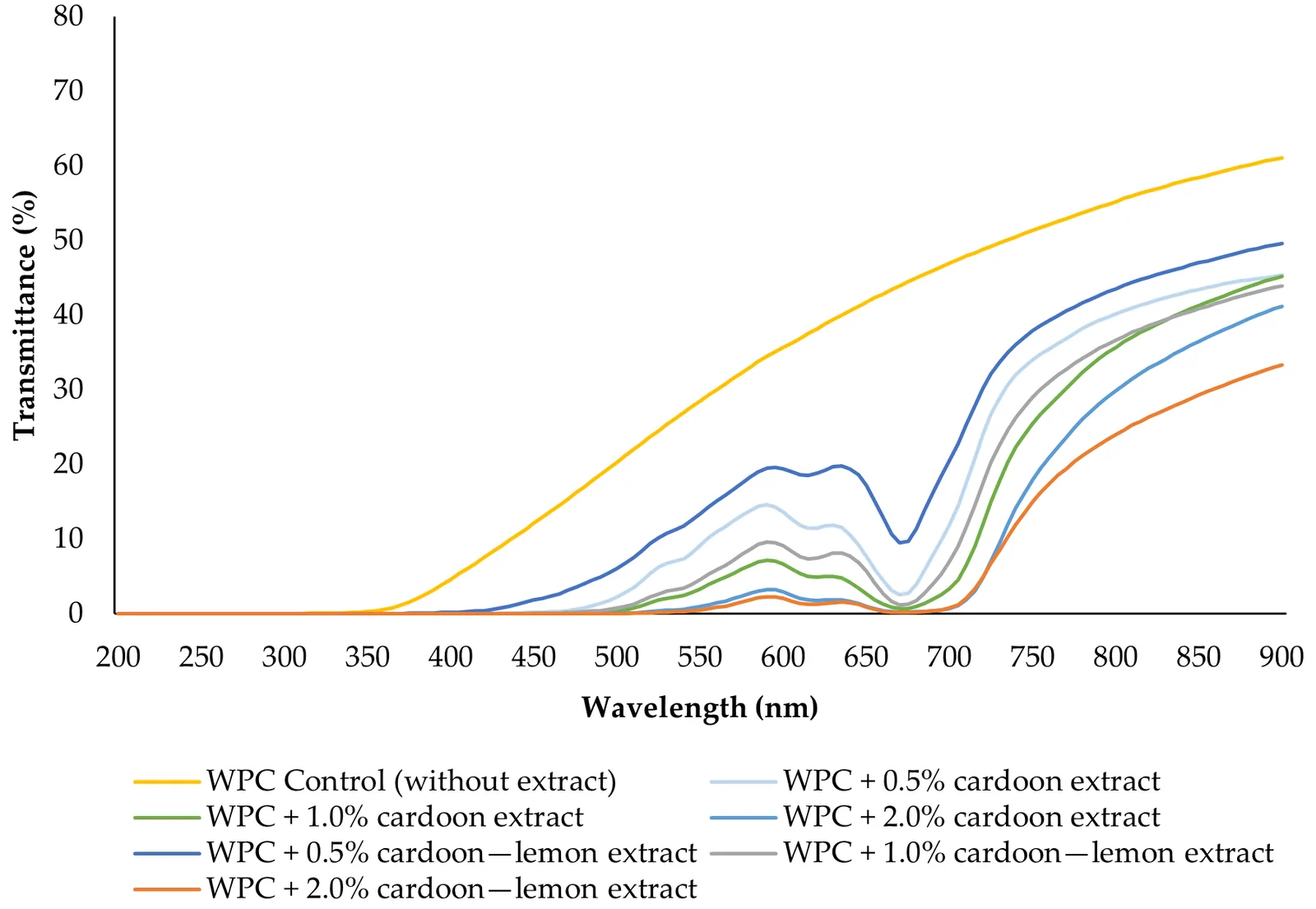
UV–VIS transparency spectra of the WPC films incorporating cardoon and cardoon–lemon extracts at different concentrations (0.5%, 1.0% and 2.0%).

The physicochemical properties of the films are presented in Table 2. The incorporation of cardoon leaf extract did not affect the film thickness compared to WPC-C. The incorporation of the cardoon–lemon extract increased the thickness at all concentrations, but with no significant difference (*p* > 0.05). According to the literature, an increase in film thickness suggests greater deposition of solids and stronger matrix–extract interactions, resulting in a less homogeneous and thicker matrix. On the other hand, a decrease in thickness indicates better dispersion and interaction of an extract with a matrix, promoting a more homogeneous distribution resulting in a less thick film [48,49]. The results for the films’ moisture content, swelling, and solubility are presented in Table 2. The addition of the extracts to the whey protein films reduced the moisture content of the films from 19.8% to 15.8%. This reduction is likely due to the phenolic compounds in the extracts, which decreased film hygroscopicity [13,14]. Following the same pattern, the swelling index also decreased with the addition of extracts. WPC-1.0C had the lowest swelling index value (7.5%). The decrease in swelling indicates that the films have improved resistance to water uptake and expansion. The active films also presented with lower solubility compared to the control film, suggesting that the extracts confer greater resistance to films in a humid environment [13,14]. WPC-1.0CL had the lowest solubility value (48.81%). Papadaki et al. [15] developed a whey protein film incorporating spent coffee grounds extract and reported an increase in the thickness of the film with the addition of the extract. The authors also reported that the addition of the extract did not affect the solubility of the films, unlike in our study, but likewise decreased their swelling index. A similar result was also observed in terms of the optical properties of films, as the addition of the extract decreased the transparency of the films [15]. Moreover, the opacity of the films increased gradually with the increase in the concentration of the extracts (Table 2). These results are in accordance with the transparency (Figure 1) assessment, where WPC-C presented with a higher transparency, which decreased with the increase in the concentration of the extracts. Overall, all the films with cardoon–lemon extract presented with higher transparency (or lower opacity) compared to the films with only cardoon extract. These results are in line with the data obtained by measuring the CIE-L* a* b* colour parameters (Table 2), in which the active films showed lower brightness (L*) values compared to WPC-C, indicating that the films became darker. Furthermore, the addition of both extracts made the films greener (lower a* values) and less yellowish (lower b* values) than WPC-C. Also, the addition of the extracts significantly (*p* < 0.05) altered the colour of the films, reducing their brightness and chromatic intensity, thus making the films more opaque and less saturated. These changes could be beneficial, as they reduce light transmission, leading to protection of food against oxidation, but may also limit product visibility, a factor that must be considered in relation to consumer expectations. The change in the optical properties (opacity, transparency and colour) observed in the whey protein films incorporating cardoon and cardoon–lemon extracts are consistent with the existing literature. For instance, an alginate–whey protein isolate presented with reduced film transparency and increased yellowness and opacity with the incorporation of curcumin [16]. Likewise, Agudelo-Cuartas et al. [17] also reported a change in the optical properties (increased opacity and L*, a* and b* coordinates) of a whey protein-based film incorporating an natamycin and α-tocopherol nanoemulsion.

Overall, the incorporation of cardoon and cardoon–lemon extracts modified the physicochemical properties of the WPC films, leading to reduced moisture, solubility and swelling; increased opacity; and noticeable colour changes, which may be beneficial for light protection in food packaging applications.

#### 3.2.2. Mechanical Properties

Table 3 presents the mechanical properties of the films. Overall, the addition of the extract reduced the tensile strength of the films, except for WPC-1.0C (0.44 ± 0.20 MPa) and WPC-2.0CL (0.51 ± 0.28 MPa). The decrease in the tensile strength indicates that there was a change in the structure of the film, making it less resistant. This reduction suggests that extract incorporation interfered with the structural cohesion of the protein matrix, weakening the intermolecular interactions and decreasing the resistance of the films to applied stress. In terms of the elongation at break, there was a decrease from 13.12 ± 3.30% (WPC-C) to 6.58 ± 0.70% (WPC-2.0C). The decrease was more pronounced in the films containing cardoon extract than in those with cardoon–lemon extract. This reflects reduced elasticity of the protein network, which may result from stronger cross-linking or reduced chain mobility caused by phenolic–protein interactions. In terms of Young’s modulus, the incorporation of extracts generally increased the Young’s modulus values, suggesting enhanced molecular interactions between the phenolic compounds and the protein matrix. This reinforcement restricted polymer chain mobility, resulting in films with greater rigidity and resistance to elastic deformation.

In summary, the extracts altered the mechanical performance of WPC films by generally reducing the tensile strength and elongation at break, while increasing the rigidity (Young’s modulus), suggesting a trade-off between flexibility and stiffness that depends on the extract type and concentration.

Comparing these results with the existing literature, it is evident that the effect of incorporating plant extracts on the mechanical properties of WPC films depends on the nature of the extract and the interactions it forms with a protein matrix. In the study by Xu et al. [19], a modified whey protein film presented with an increase in tensile strength and stiffness, followed by a decrease in elongation at break, attributed to the formation of a denser and more cohesive protein network. Similarly, Andrade et al. [12] incorporated *Fucus vesiculosus* extract into whey protein films, which led to a slight tendency towards increased tensile strength and modulus of elasticity, although no statistically significant differences were observed. However, in the present study, the incorporation of cardoon leaf and lemon peel extracts generally led to a decrease in the tensile strength and elongation at break, and an increase in Young’s modulus. These results may suggest that, while the phenolic compounds restricted the mobility of the polymer chains, thereby increasing the stiffness of the films, they also compromised the cohesion of the protein matrix, reducing its mechanical strength. These mechanical changes are consistent with the structural observations obtained by SEM and ATR-FTIR. The increased surface roughness and heterogeneity observed in the cardoon-containing films suggest disruption of the protein network, while the FTIR spectra confirm the incorporation of extract-derived compounds into the WPC matrix. These interactions may have restricted polymer chain mobility, increasing rigidity while reducing matrix cohesion and flexibility.

#### 3.2.3. Barrier Properties

The WVP of the films was assessed and the results are presented in Table 3. WPC-C presented with a WVP of 11.9 ± 1.49 × 10^−10^ g/(s.m.Pa). WPC-0.5C presented with almost the same value (13.40 ± 0.19 × 10^−10^ g/(s.m.Pa)), indicating that the extract did not improve the barrier properties. WPC-1.0C presented with the lowest WVP value (4.05 ± 0.26 × 10^−10^ g/(s.m.Pa)) of all the studied films. This result means this film had a more compact and homogeneous matrix formation that restricted the film’s WVP more effectively. On the other hand, the WVP of WPC-2.0C increased significantly (34.76 ± 4.49 × 10^−10^ g/s.m.Pa) (*p* < 0.05), indicating that the increase in extract concentration caused an increase in film porosity, thus leading to a higher WVP. The films with cardoon–lemon extract displayed a dose-dependent effect, as the WVP decreased with the increase in the concentration of the extract. The highest WVP was found for WPC-0.5CL (16.82 ± 5.67 × 10^−10^ g/s.m.Pa), indicating that the addition of the extract at this concentration impaired the film barrier’s properties. WPC-1.0CL (12.13 ± 0.19 × 10^−10^ g/s.m.Pa) presented with similar results as those of the control (11.9 ± 1.49 × 10^−10^ g/s.m.Pa), suggesting a better interaction between the extract and the protein matrix. Finally, WPC-2.0CL, unlike WPC-2.0C, presented with the lowest value of WVP (10.96 ± 0.12 × 10^−10^ g/s.m.Pa). This suggests that a higher concentration of the extract was more efficient at preventing water exchange, which may be due to compounds present in the lemon peel that reduced the porosity of the matrix. The variability in the WVP values among formulations is partly related to differences in film thickness; however, as all WVP calculations were normalised using the thickness of the same specimens tested, the observed variations mainly reflect compositional and microstructural changes induced by the extracts rather than thickness alone. Mirpoor et al. [50] produced a film with seed oil cake protein and cardoon leaf-derived extracts, and observed an improvement in the WVP at lower concentrations of the extract, but the opposite at higher concentrations. Alvarez-Perez et al. [18] also observed a decrease in the WVP value with the addition of tarbush polyphenols to a whey protein–candelilla wax film.

#### 3.2.4. Thermogravimetric Analysis (TGA)

**Figure 2:**
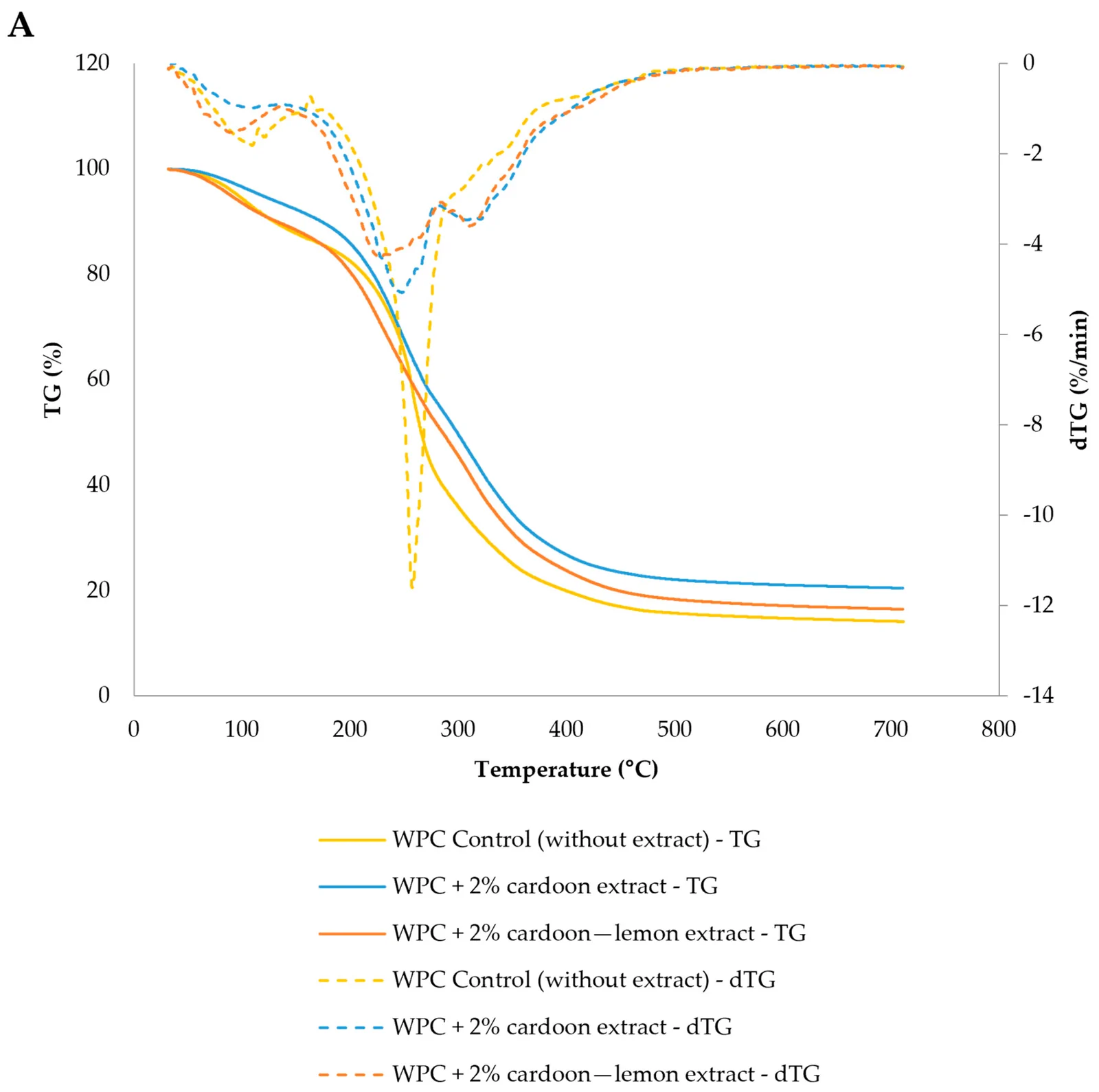

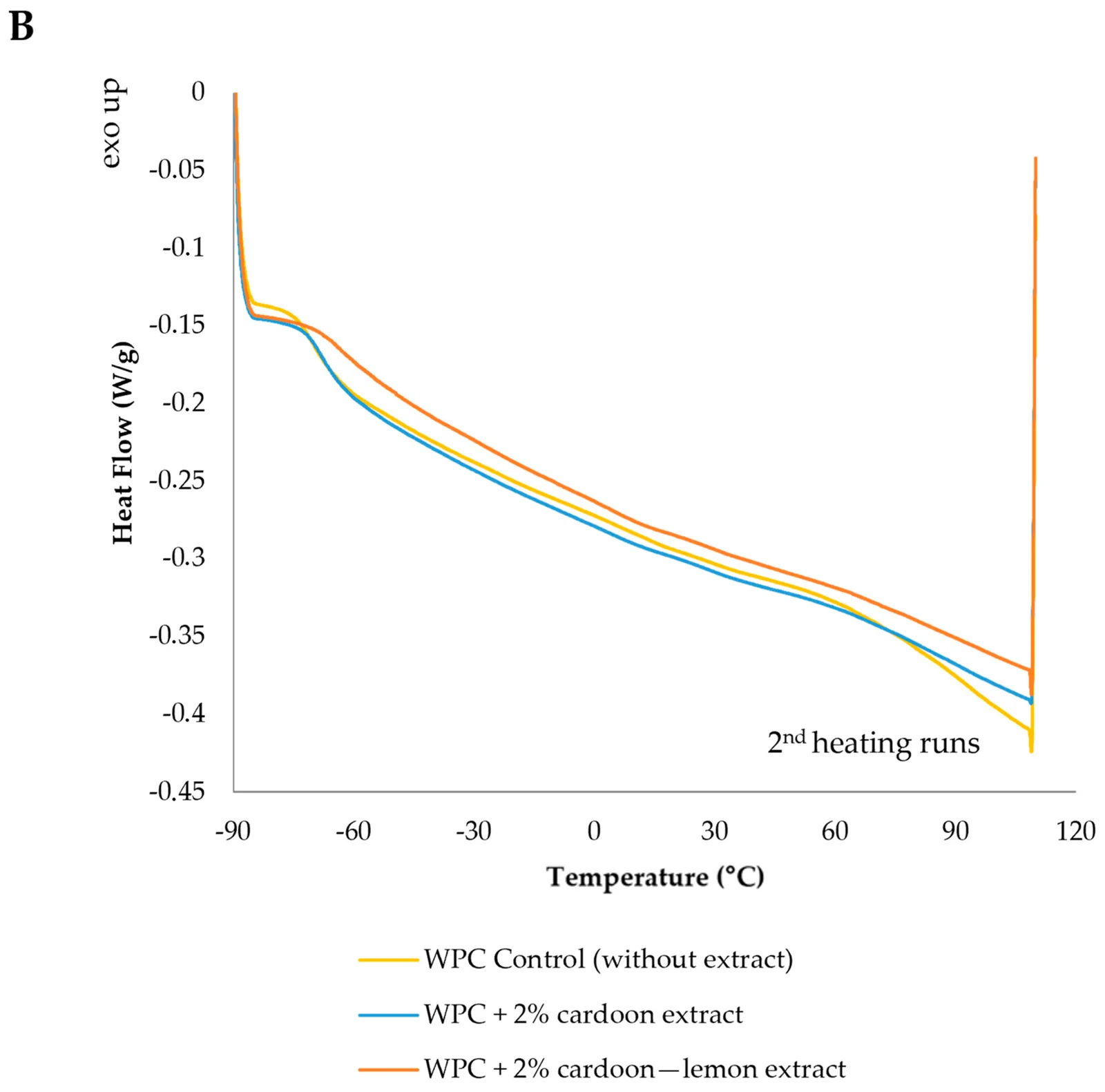
Thermal analysis of WPC films incorporating cardoon and cardoon–lemon extracts. (**A**) Combined thermogravimetric (TG, solid line) and derivative thermogravimetric (dTG, dashed line) curves for control film (WPC-C), film with 2.0% cardoon extract (WPC-2.0C), and film with 2.0% cardoon–lemon extract (WPC-2.0CL). TG curves show mass loss, while dTG curves highlight decomposition steps (Tinf) and differences in thermal stability. (**B**) Differential scanning calorimetry (DSC) thermograms of same films showing glass transition temperatures (Tg onset).

The thermogravimetric analysis (TG/dTG) revealed differences in the thermal behaviour of whey protein concentrate-based films related to the incorporation of natural extracts (Table 4, Figure 2A). WPC-C exhibited two main mass loss events: the first, between 41 °C and 159 °C, was attributed to the removal of adsorbed water and volatile compounds; and the second, between 159 °C and 518 °C, was associated with the degradation of protein chains and the consequent formation of carbonised residues. The main decomposition occurred at 256 °C (T_inf_—inflection temperature of decomposition), and the residual mass was approximately 16% of the initial mass, reflecting a predominantly proteinaceous organic structure with relatively low thermal stability.

Incorporation of 2.0% cardoon leaf extract (WPC-2.0C) altered the decomposition profile, leading to three distinct thermal stages. The first event, between 43 °C and 135 °C, showed a lower mass loss (6.35%), suggesting a reduced free water content (in agreement with the lower moisture content measured) and higher matrix cohesion, possibly due to the hydrogen bond interactions between the phenolic compounds and protein chains. The second event, between 134 °C and 281 °C, corresponded to the main degradation step (Δm = 38.18%), during which T_inf_ shifted to 265 °C, indicating a slight improvement in thermal stability. The third event, between 281 °C and 568 °C, exhibited further mass loss (34.27%), with T_inf_ at 306 °C, confirming the presence of carbonised residues and partially stabilised phenolic compounds. These results indicate that the cardoon leaf extract conferred enhanced overall thermal resistance to the film, promoting a more gradual decomposition.

WPC-2.0CL displayed three thermal stages. The first, between 34 °C and 135 °C, showed a mass loss of 10.11%, slightly higher than that observed in WPC-2.0C, likely due to the volatile citrus compounds. The second event, between 135 °C and 284 °C, was the main degradation process (Δm = 39.32%), with T_inf_ at 234 °C, followed by a third stage between 284 °C and 587 °C, with additional mass loss of 33.03% and T_inf_ at 308 °C. Although the second peak occurred at a slightly lower temperature than in WPC-2.0C, the final decomposition extended to higher temperatures, reflecting a more complex and heterogeneous matrix where the phenolic compounds and organic acids interacted complementarily with the whey proteins, promoting the formation of partially carbonised and thermally more stable structures.

#### 3.2.5. Differential Scanning Calorimetry (DSC)

The DSC analysis (Figure 2B) allowed for the evaluation of the glass transition temperature (T_g_) of the films and provided information on the segmental mobility of the protein chains. In the first heating run, only water evaporation was detected. The heat flow step associated with the glass transition was not observed, as it becomes visible within the measured temperature range only after substantial dehydration, revealing a T_g_ below −90 °C, which is characteristic of hydrated biopolymeric systems with high molecular flexibility [51]. The shift of the glass transition to higher temperatures upon dehydration further confirms the plasticising effect of water in such systems [52]. In the following heating run, all films exhibited cryogenic T_g_ values: WPC-C showed a T_g_ onset of −74.41 °C; incorporation of 2% cardoon extract (WPC-2.0C) resulted in a slight increase in T_g_ to −71.86 °C, while WPC-2.0CL reached −69.16 °C.

The progressive increase in T_g_ (less negative values), which is also evidenced by the derivative plot against temperature of the heat flow (Figure 2B), indicates a reduction in segmental mobility of the protein chains, attributed to the presence of phenolic compounds and organic acids that established hydrogen bonds and hydrophobic interactions with the protein matrix [51,53]. This restriction of molecular motion, observed mainly in the sample with lemon peel extract, translated into a greater structural cohesion and modest enhanced thermal stability, consistent with a broader and slightly increased T_g_.

In summary, the DSC confirms that the natural extracts promoted increased rigidity and thermal stability of the films, complementing the observations obtained through the T_g_/dT_g_ analysis.

#### 3.2.6. Scanning Electron Microscopy (SEM)

**Figure 3:**
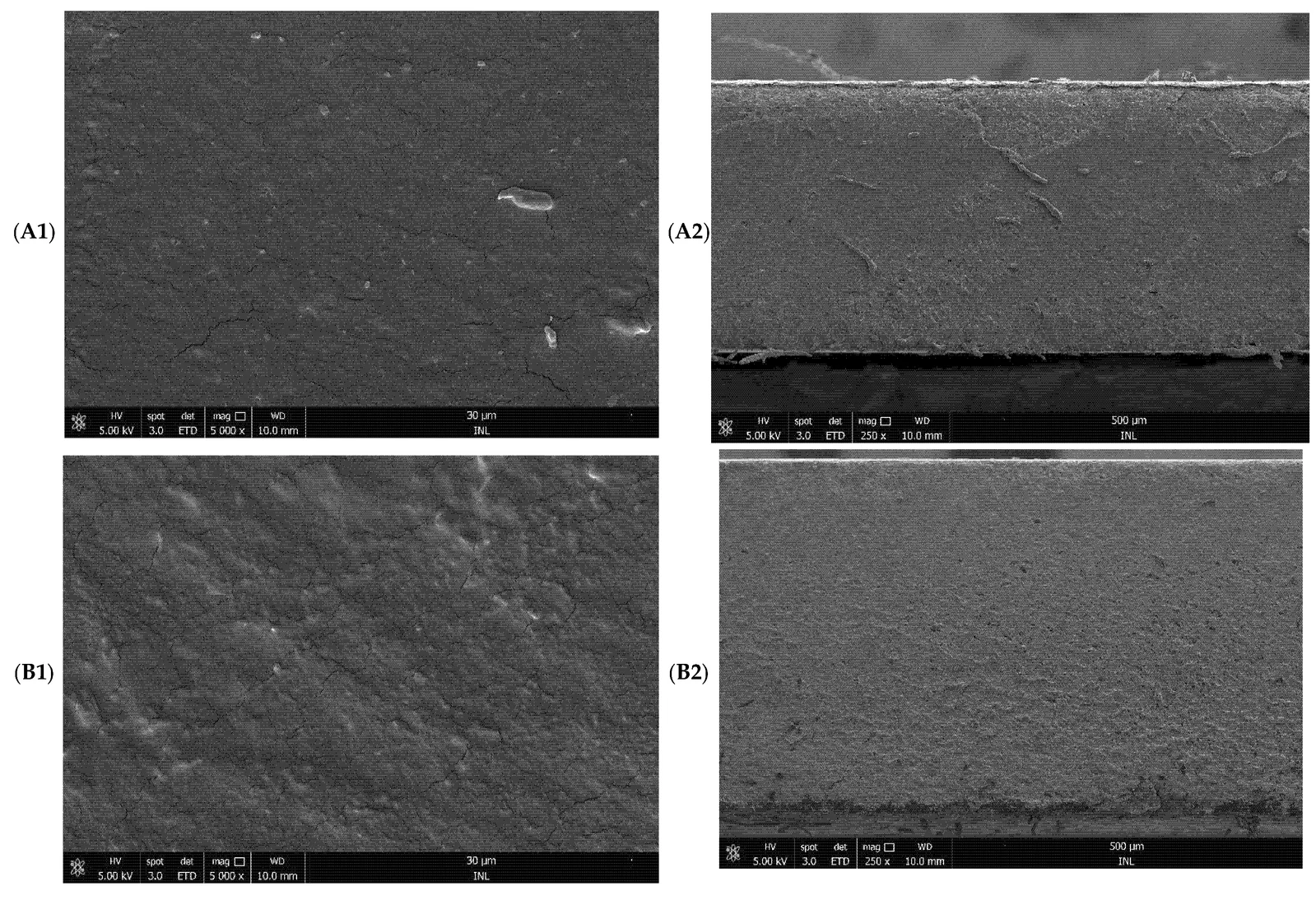

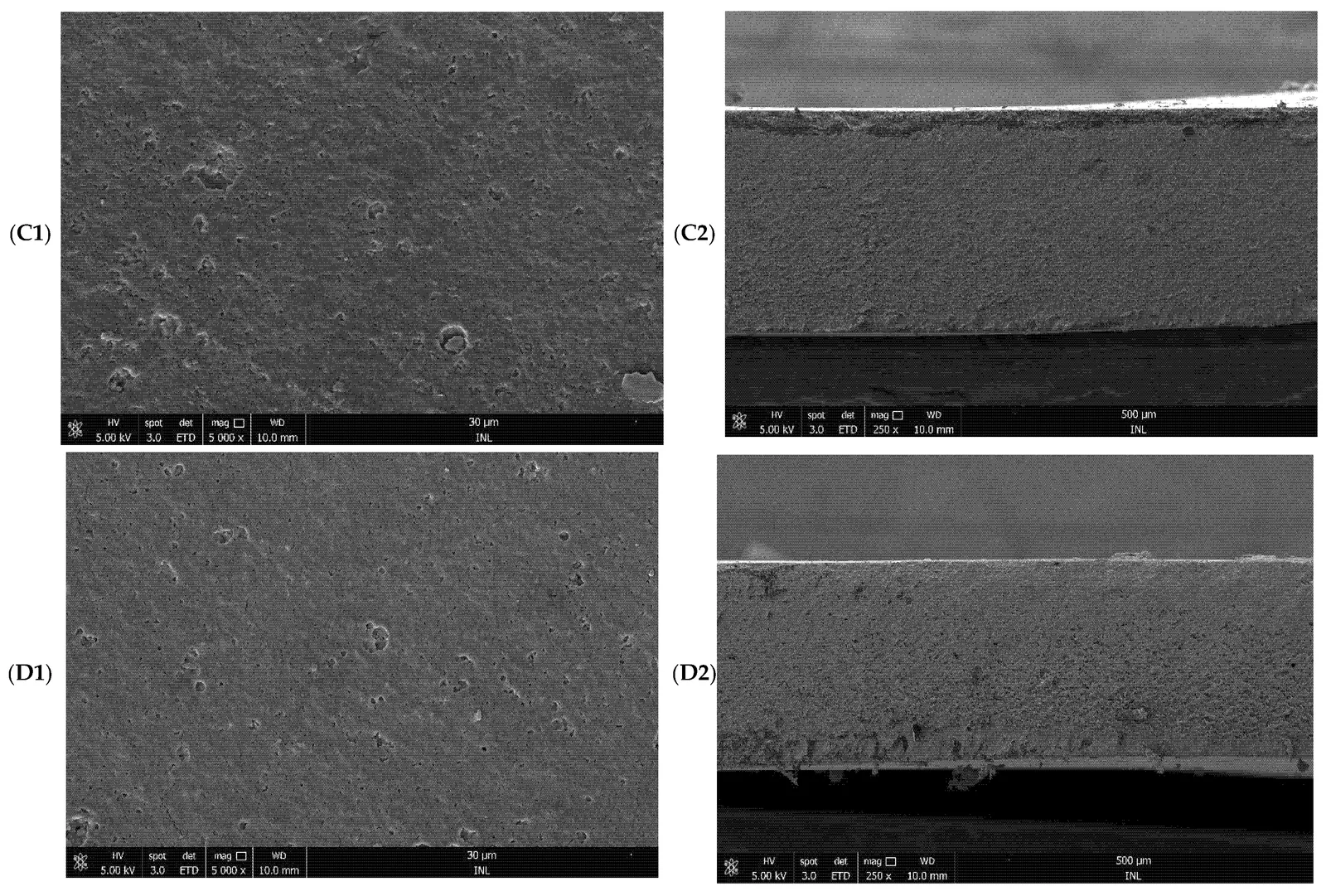

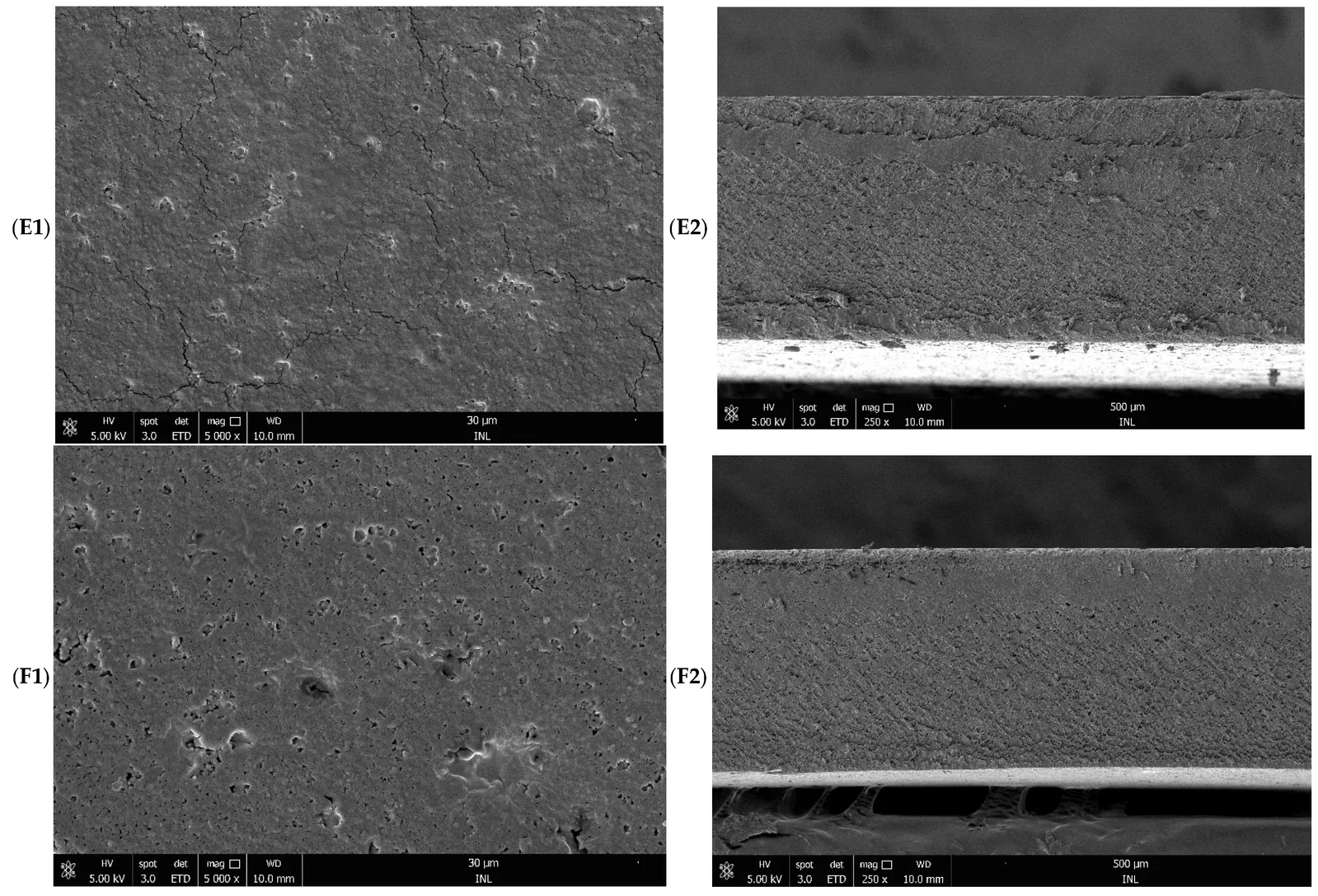

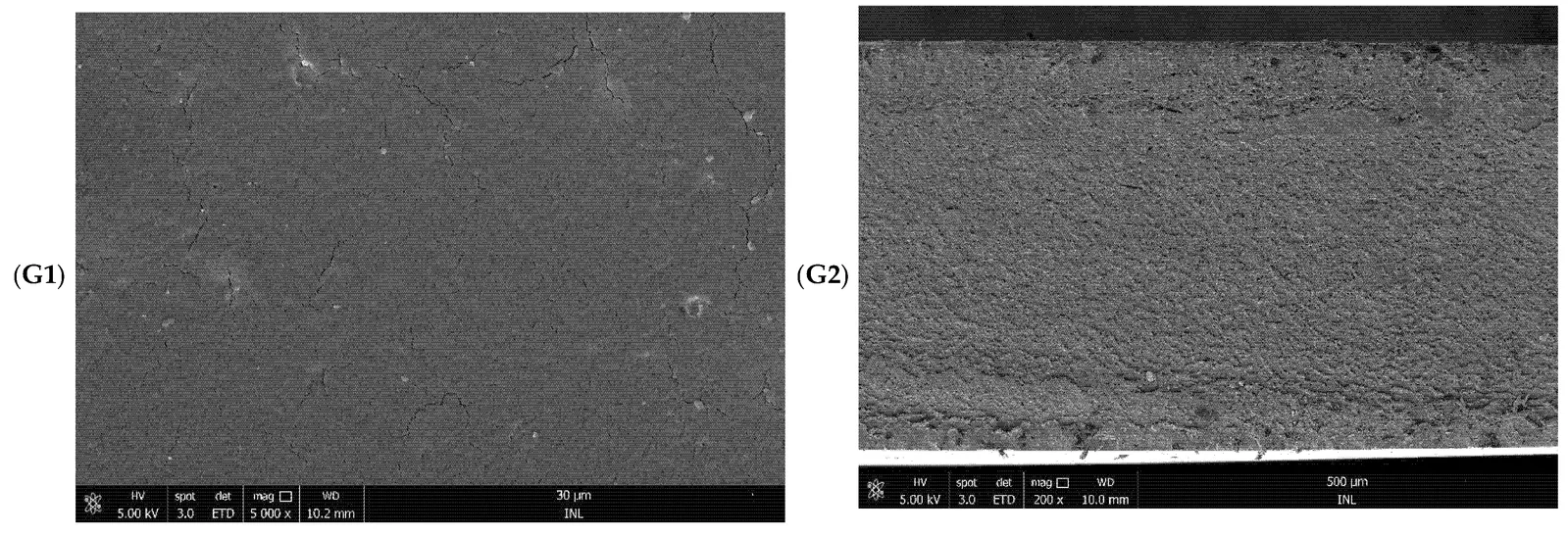
Images obtained by SEM of the WPC films incorporating cardoon and cardoon–lemon extracts at different concentrations (0.5%, 1.0% and 2.0%). (**A**–**G**) WPC-C (**A**), WPC-0.5C (**B**), WPC-1.0C (**C**), WPC-2.0C (**D**), WPC-0.5CL (**E**), WPC-1.0CL (**F**), and WPC-2.0CL (**G**). The “1” corresponds to surface images (**A1**–**G1**), while “2” refers to cross-section images (**A2**–**G2**). Images were acquired at 5.00 kV, spot size 3.0, ETD detector (secondary electrons), at 5000× magnification, 10.0 mm working distance, and 30 µm scale bar for surface images; and at 250× magnification with a 500 µm scale bar for cross-section images.

The morphological analysis of the surface and cross-sectional structures of the films incorporating cardoon leaf and cardoon leaf–lemon peel extracts revealed structural changes associated with the addition of the extracts at different concentrations (0.5%, 1.0%, and 2.0%). The WPC-C exhibited a smooth and homogeneous surface (Figure 3(A1,A2)), typical of pure WP matrices [54]. In contrast, the films containing cardoon leaf extract showed progressively increased surface roughness and heterogeneity with rising extract concentration (Figure 3B–D), likely due to the presence of phenolic compounds and plant residues that disrupted the protein network [54]. The films with cardoon leaf–lemon peel extract displayed a more compact and uniform surface (Figure 3E–G), suggesting improved compatibility between the bioactive components and the protein matrix. This may have resulted from the complementary interactions between the phenolic compounds present in the extracts, enhancing molecular cohesion [54]. The cross-sectional images (Figure 3(A2–G2)) confirm dense and cohesive internal structures across all formulations, with only minor variations. WPC-2.0CL exhibited the most homogeneous dispersion of the extract within the protein matrix.

#### 3.2.7. Attenuated Total Reflectance Fourier-Transform Infrared Spectroscopy (ATR-FTIR)

**Figure 4:**
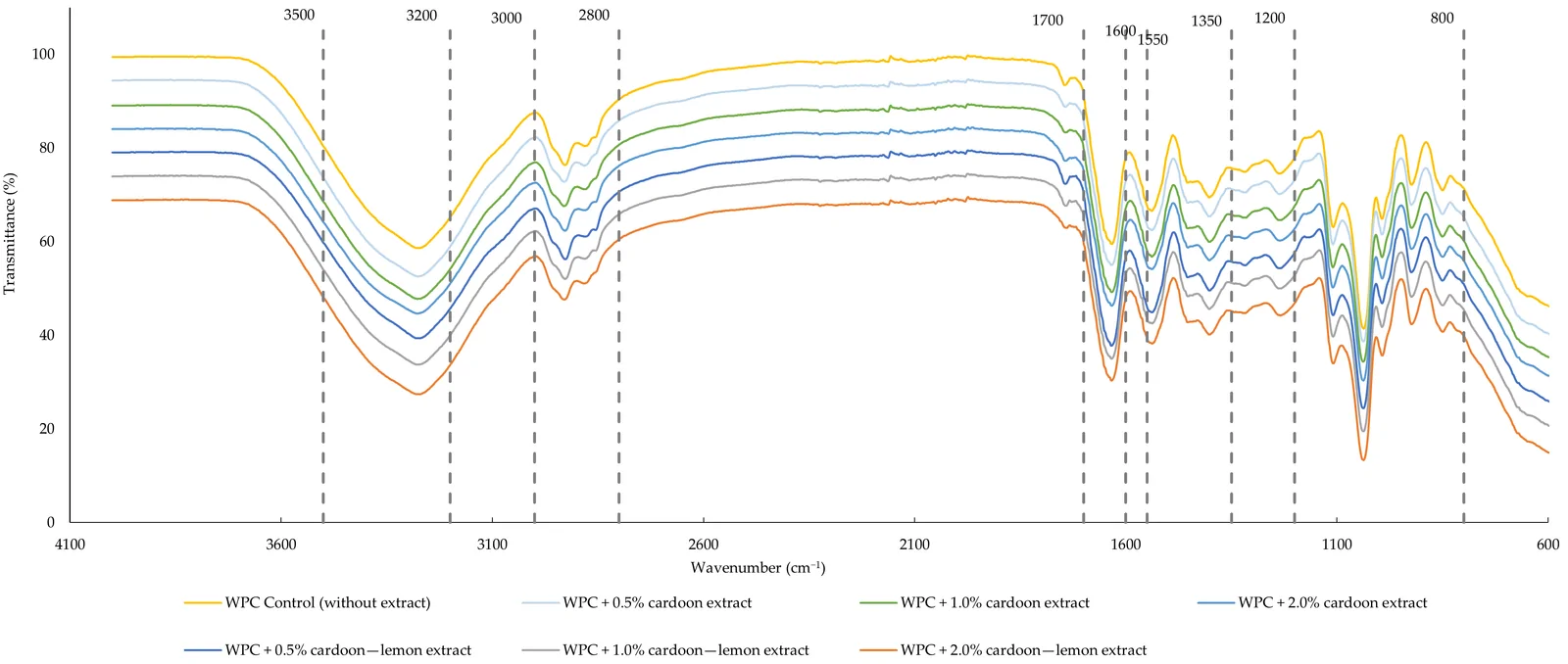
ATR–FTIR spectra of the WPC films incorporated with cardoon and cardoon–lemon extracts at different concentrations (0.5%, 1.0% and 2.0%).

The molecular composition of the films was analysed by ATR-FTIR and the results are shown in Figure 4. All tested films displayed characteristic absorption peaks of WPC between 3200 and 3500 cm^−1^, corresponding to free and bound O-H and N-H groups, and between 2800 and 3000 cm^−1^, corresponding to C-H stretching vibrations [17,19]. The peaks between 1600 and 1700 cm^−1^ were identified as C=O stretching and C-N bonding (Amide I), those between 1500 and 1550 cm^−1^ to C-O stretching (Amide II), and those from 1200 to 1350 cm^−1^ to C-N stretching and N-H bonding (Amide III) [17,19]. An additional absorption band between 800 and1200 cm^−1^ was attributed to the incorporation of the extracts and glycerol into the protein matrix [16,17,19], confirming the successful integration of these compounds. Similar ATR–FTIR patterns have been reported for other whey protein films, with slight variations depending on the type of active compound incorporated [16,17,19,20,50].

Overall, the ATR–FTIR analysis confirms the successful incorporation of the cardoon and cardoon–lemon extracts into the whey protein matrix, with spectral patterns consistent with previously reported whey protein films and slight variations depending on the type and concentration of the active compound.

## 4. Conclusions

Cardoon leaves have a notable nutritional profile, being rich in protein (21.2 ± 0.4 g/100 g), dietary fibre (43.9 ± 1.8 g/100 g), essential amino acids and micronutrients (Ca, Mg, K, P), and bioactive compounds (chlorogenic acid, luteolin and apigenin). Overall, the nutritional profile of the cardoon leaves improved after drying, with higher concentrations of some nutrients, such as dietary fibre, minerals, amino acids, and fatty acids. This improvement is primarily attributed to the significant reduction in water content and the resistance of these compounds to thermal degradation during the drying process. Thus, the valorisation of agro-industrial by-products, such as cardoon leaves, shows promise for application in food and active food packaging, particularly as coatings or inner layers in multilayer packaging structures.

The incorporation of cardoon and cardoon–lemon extracts into whey protein concentrate for the development of potentially active films was successful. Although some limitations in their mechanical properties (increased rigidity) were observed, likely due to the interactions between the phenolic compounds and the protein matrix reducing chain mobility, the improvements in the barrier and optical properties (lower WVP and decrease in transparency) indicate that these films represent a promising and innovative strategy for the valorisation of cardoon leaves. Therefore, the results support the potential applicability of these plants extracts in the production of sustainable food packaging, in line with the principles of circular economy and reduction in agro-industrial waste. Nonetheless, further research should include analysis of the antioxidant and antimicrobial properties of the films to confirm their functional properties and elucidate the mechanisms underlying their potential effects, alongside a Life Cycle Assessment and economic viability studies to further evaluate the sustainability and scalability of the process.

## Figures and Tables

**Table 1:** Nutritional profile of fresh and dry cultivated cardoon leaves.

	Fresh Leaves	Dry Leaves
Nutritional Composition		
Moisture (g/100 g)	81.3 ± 0.3	7.4 ± 0.1
Ash (g/100 g DW)	12.7 ± 0.0	13.7 ± 0.2
Total Protein (g/100 g DW) *	22.3 ± 0.6	21.2 ± 0.4
Total Fat (g/100 g DW)	1.1 ± 0.0	2.0 ± 0.1
Total Sugars (g/100 g DW)	5.0 ± 0.0	3.7 ± 0.0
Total Dietary Fibers (g/100 g DW) *	42.1 ± 2.5	43.9 ± 1.8
Carbohydrates (g/100 g DW)	16.9 ± 0.5	15.4 ± 1.1
Energy (KJ/100 g DW)	1127 ± 4	1111 ± 9
Energy (Kcal/100 g DW)	270 ± 1	267 ± 2
Fatty Acid Profile (g/100 g DW)		
C10:0	ND	0.01 ± 0.00
C12:0	ND	0.02 ± 0.00
C14:0	ND	0.01 ± 0.00
C16:0	0.05 ± 0.00	0.09 ± 0.02
C18:0	ND	0.03 ± 0.00
C18:1*c*	0.11 ± 0.01	0.01 ± 0.00
C18:2*c* (*n*6)	0.21 ± 0.01	0.11 ± 0.00
C18:3*c* (*n*3)	0.06 ± 0.01	0.11 ± 0.01
C18:3*c* (*n*6)	ND	0.15 ± 0.02
C20:0	ND	0.04 ± 0.00
C20:3 (*n*6)	ND	0.30 ± 0.01
C20:4 (*n*6)	ND	0.01 ± 0.00
C22:0	0.17 ± 0.01	0.02 ± 0.00
C24:0	0.35 ± 0.03	0.76 ± 0.04
C24:1	ND	0.01 ± 0.00
SFAs	0.59 ± 0.05	4.86 ± 0.05
MUFAs *	0.11 ± 0.01	0.13 ± 0.03
PUFAs	0.32 ± 0.04	4.22 ± 0.05
Mineral Profile (mg/100 g DW)		
Phosphorus	367.8 ± 21.2	535.1 ± 9.0
Magnesium	319.8 ± 2.7	359.3 ± 8.3
Calcium	1740.7 ± 23.5	1666.5 ± 15.1
Potassium *	3668.1 ± 106.6	3733.9 ± 63.7
Sodium	199.2 ± 1.8	272.1 ± 5.7
Manganese	7.6 ± 0.4	5.2 ± 0.1
Iron	22.6 ± 0.8	10.9 ± 0.2
Zinc	2.4 ± 0.2	3.4 ± 0.1
Copper	0.2 ± 0.0	0.7 ± 0.0
Amino Acid Profile (mg/100 g DW)		
Essential		
Histidine	563.9 ± 25.6	462.8 ± 3.5
Isoleucine *	754.1 ± 30.6	750.9 ± 12.0
Leucine	1270.1 ± 61.2	1615.0 ± 34.6
Lysine	458.9 ± 20.6	1053.2 ± 40.6
Methionine	433.5 ± 16.7	215.8 ± 8.1
Phenylalanine	1235.8 ± 43.0	1162.6 ± 11.1
Threonine	644.8 ± 25.6	754.3 ± 55.4
Valine *	1174.4 ± 46.7	1157.2 ± 14.6
Total essential amino acids	7433.24	8226.10
Non-essential		
Alanine	2343.1 ± 100.4	1308.4 ± 4.1
Arginine	861.4 ± 35.6	832.0 ± 24.4
Aspartic acid	1060.3 ± 47.3	3593.6 ± 104.5
Cysteine	ND	ND
Glutamic acid	1563.2 ± 67.2	2358.7 ± 164.7
Glycine	897.8 ± 39.4	1054.3 ± 20.3
Proline	1189.5 ± 50.9	2168.5 ± 125.3
Serine	750.0 ± 31.2	821.5 ± 70.7
Tyrosine	1119.7 ± 46.6	821.4 ± 54.2
Total non-essential amino acids	8887.21	11,904.19
Carotenoids (mg/100 g DW)		
α-carotene	ND	ND
β-carotene	23.51 ± 0.42	8.53 ± 0.19
*cis*-β-carotene *	2.89 ± 0.02	2.81 ± 0.35
β-cryptoxanthin	0.53 ± 0.03	0.25 ± 0.00
Lycopene	ND	ND
Lutein	6.41 ± 0.19	0.25 ± 0.01
Zeaxantine	ND	ND
Vitamin A (mg/100 g DW)	ND	ND
Vitamin E (mg/100 g DW)	4.92 ± 0.01	1.73 ± 0.00

DW—dry weight; ND—not detected; SFAs—saturated fatty acids; MUFAs—monosaturated fatty acids; PUFAs—polysaturated fatty acids. Results are presented as means ± standard deviation of three replicates. All values are significantly different between samples (*p* < 0.05). * Indicates results that are not significantly different.

**Table 2:** Physicochemical properties of the WPC films incorporating cardoon and cardoon–lemon extracts at different concentrations (0.5%, 1.0% and 2.0%).

Film	Thickness (µm)	Moisture Content (%)	Swelling (%)	Solubility (%)	Opacity (mm^−1^)	L*	a*	b*	Hue Angle (°)	Chroma
WPC Control (without extract)	520.87 ± 59.57 ^a^	19.79 ± 1.07 ^a^	20.38 ± 0.55 ^a^	57.35 ± 0.72 ^a^	0.87 ± 0.05 ^d^	82.23 ± 1.77 ^a^	3.82 ± 0.82 ^a^	33.89 ± 3.28 ^a^	1.46 ± 0.02 ^a^	34.11 ± 3.32 ^a^
WPC + 0.5% cardoon extract	519.27 ± 72.00 ^a^	16.97 ± 0.75 ^a^	14.83 ± 1.50 ^bc^	55.56 ± 1.49 ^ab^	1.71 ± 0.27 ^c^	39.35 ± 2.55 ^c^	0.44 ± 0.28 ^d^	7.19 ± 0.11 ^bc^	1.51 ± 0.04 ^a^	7.21 ± 0.12 ^bc^
WPC + 1.0% cardoon extract	496.93 ± 52.99 ^a^	16.59 ± 18.18 ^a^	7.45 ± 2.06 ^d^	51.71 ± 3.37 ^abc^	2.40 ± 0.10 ^a^	32.25 ± 1.99 ^d^	0.36 ± 0.19 ^d^	7.19 ± 0.11 ^bc^	1.51 ± 0.02 ^a^	5.69 ± 1.18 ^bc^
WPC + 2.0% cardoon extract	678.07 ± 177.54 ^a^	15.79 ± 0.90 ^a^	12.26 ± 0.72 ^cd^	52.08 ± 0.69 ^abc^	2.52 ± 0.08 ^a^	29.90 ± 0.64 ^d^	0.57 ± 0.24 ^cd^	5.68 ± 1.17 ^c^	1.19 ± 0.06 ^c^	1.53 ± 0.69 ^c^
WPC + 0.5% cardoon–lemon extract	598.80 ± 175.58 ^a^	16.94 ± 1.94 ^a^	19.79 ± 1.72 ^ab^	50.07 ± 1.00 ^abc^	1.28 ± 0.13 ^cd^	48.75 ± 6.92 ^b^	1.44 ± 0.07 ^bc^	27.76 ± 8.06 ^a^	1.51 ± 0.02 ^a^	27.80 ± 8.05 ^a^
WPC + 1.0% cardoon–lemon extract	613.67 ± 77.30 ^a^	20.26 ± 1.22 ^a^	17.90 ± 1.93 ^abc^	48.81 ± 2.52 ^c^	1.73 ± 0.21 ^bc^	34.82 ± 1.93 ^cd^	1.50 ± 0.37 ^b^	10.44 ± 2.96 ^b^	1.43 ± 0.01 ^a^	10.55 ± 2.98 ^b^
WPC + 2.0% cardoon–lemon extract	808.67 ± 88.16 ^a^	18.62 ± 2.45 ^a^	15.54 ± 1.34 ^abc^	49.47 ± 3.30 ^bc^	2.16 ± 0.03 ^ab^	29.96 ± 1.61 ^d^	0.78 ± 0.28 ^bcd^	2.80 ± 0.41 ^bc^	1.30 ± 0.08 ^b^	2.92 ± 0.43 ^bc^

Results are presented as means ± standard deviation. Different letters represent results with significant differences (*p* < 0.05).

**Table 3:** Mechanical and barrier properties of the WPC films incorporating with cardoon and cardoon–lemon extracts at different concentrations (0.5%, 1.0% and 2.0%).

Film	Mechanical Properties			Barrier Properties
	Tensile Strength (MPa)	Elongation at Break (%)	Young’s Modulus (MPa)	WVP (10^−10^ g/s.m.Pa)
WPC Control (without extract)	0.34 ± 0.06 ^c^	13.12 ± 3.30 ^a^	0.06 ± 0.01 ^e^	11.90 ± 1.49 ^bc^
WPC + 0.5% cardoon extract	0.31 ± 0.09 ^d^	9.08 ± 2.52 ^d^	0.07 ± 0.01 ^e^	13.40 ± 0.19 ^bc^
WPC + 1.0% cardoon extract	0.44 ± 0.20 ^b^	9.31 ± 4.77 ^c^	0.10 ± 0.02 ^ab^	4.05 ± 0.26 ^d^
WPC + 2.0% cardoon extract	0.23 ± 0.04 ^e^	6.58 ± 0.70 ^f^	0.08 ± 0.03 ^cd^	34.76 ± 4.49 ^a^
WPC + 0.5% cardoon–lemon extract	0.23 ± 0.01 ^e^	11.26 ± 3.98 ^b^	0.09 ± 0.00 ^bc^	16.82 ± 5.67 ^b^
WPC + 1.0% cardoon–lemon extract	0.32 ± 0.11 ^d^	10.26 ± 3.98 ^b^	0.06 ± 0.01 ^e^	12.13 ± 0.19 ^bc^
WPC + 2.0% cardoon–lemon extract	0.51 ± 0.28 ^a^	8.40 ± 2.73 ^e^	0.11 ± 0.03 ^a^	10.96 ± 0.12 ^c^

Results are presented as means ± standard deviation. Different letters represent results with significant differences (*p* < 0.05). WVP—water vapour permeability.

**Table 4:** Thermogravimetric analysis (TG/dTG) of the WPC films incorporating cardoon and cardoon–lemon extracts at different concentrations (0.5%, 1.0% and 2.0%).

Film	Step	Temperature Range (°C)	Δm (%)	T_inf_ (°C)	Residual Mass (%)
WPC Control (without extract)	1	41.2–158.8	12.91	112.5	15.71
	2	158.8–518.5	71.38	256.2	
WPC + 2.0% cardoon extract	1	43.2–134.9	6.36	103.9	21.18
	2	134.1–280.9	38.18	265.2	
	3	280.5–567.7	34.27	305.8	
WPC + 2.0% cardoon–lemon extract	1	33.8–135.4	10.11	86.5	17.53
	2	135.2–283.8	39.32	233.5	
	3	284.3–586.8	33.03	308.0	

Δm—mass loss; T_inf_—inflection temperature of decomposition.

## Data Availability

The original contributions presented in this study are included in the article. Further inquiries can be directed to the corresponding author.
